# Relationship of ASPECTS Lesion Topography with Clinical Outcomes in Acute Ischemic Stroke Treated with Endovascular Thrombectomy: A Single-Center Cohort Study

**DOI:** 10.3390/diagnostics16060822

**Published:** 2026-03-10

**Authors:** Hilmiye Tokmak, Hamza Özer, Muhammed Nur Öğün

**Affiliations:** 1 Department of Neurology, Niğde Training and Research Hospital, Niğde 51100, Turkey; 2 Department of Radiology, Faculty of Medicine, Bolu Abant İzzet Baysal University, Bolu 14030, Turkey; hozermd@gmail.com; 3 Department of Neurology, Faculty of Medicine, Bolu Abant İzzet Baysal University, Bolu 14030, Turkey; dr.mogun@gmail.com

**Keywords:** acute ischemic stroke, endovascular thrombectomy, ASPECTS, lesion localization, clinical outcome

## Abstract

**Objective**: Although an Alberta Stroke Program Early CT Score (ASPECTS) < 7 is known to be associated with poor clinical outcomes in patients with acute ischemic stroke (AIS), the relationship between regional differences in infarct location within the ASPECTS territory and clinical outcome has not been fully clarified. The aim of this study is to evaluate the association between infarct area localization and clinical outcomes in AIS patients with large vessel occlusion and to investigate whether these regional patterns can be used to predict prognosis independently of the total ASPECTS. **Methods**: In this retrospective, single-center study, patients with acute ischemic stroke who had undergone non-contrast brain CT prior to endovascular thrombectomy between January 2020 and July 2023 and were found to have internal carotid artery (ICA) and/or middle cerebral artery (MCA) M1 segment occlusion were included. Patients with a premorbid modified Rankin Scale (mRS) score of 0–2 were eligible for inclusion. Patients with unavailable imaging or clinical follow-up data were excluded. Clinical outcomes were assessed using the modified Rankin Scale at 90 days. An mRS score of 0–2 was defined as a good clinical outcome, whereas an mRS score of 3–6 was defined as a poor clinical outcome. **Results**: A total of 283 patients were included (median age 73 years; 57.2% female), of whom 147 (51.9%) achieved a good clinical outcome. The poor outcome group had higher NIHSS scores and lower total ASPECTS values (both *p* < 0.001). In the regional analysis, involvement of the internal capsule (32.4% vs. 4.1%; *p* < 0.001) and ASPECTS M1 region (26.5% vs. 10.2%; *p* < 0.001) was associated with poor outcome. In multivariable analysis, internal capsule involvement (adjusted odds ratio [aOR] ≈ 12) and M1 region involvement (aOR ≈ 2.7) remained independent predictors. In the subgroup with successful recanalization, only internal capsule involvement remained an independent predictor (aOR ≈ 19). In hemisphere-stratified analyses, M1 involvement in the right hemisphere and internal capsule involvement in the left hemisphere were independently associated with poor outcome. **Conclusions**: The prognostic contribution of individual ASPECTS regions is not uniform in patients with acute ischemic stroke undergoing endovascular thrombectomy (EVT). In particular, involvement of the internal capsule and the M1 region shows a strong association with poor clinical outcome independent of the total ASPECTS. However, these findings suggest that regional localization alone is not sufficient for EVT patient selection. Further large-scale, multicenter studies are needed to clarify the role of regional ASPECTS assessment in clinical decision-making.

## 1. Introduction

Despite advances in early diagnosis and treatment options, acute ischemic stroke (AIS) remains one of the leading causes of death and long-term disability worldwide [1,2,3]. In recent years, continuous improvements in devices and techniques used in EVT have led to higher recanalization rates, particularly in strokes caused by large vessel occlusion. However, even when successful vessel recanalization is achieved, clinical outcomes are not always favorable, and despite similar treatment approaches, some patients continue to live with severe disability [4,5,6]. This uncertainty in clinical course has driven researchers to seek reliable markers that can support accurate patient selection in the acute phase and more precisely predict clinical outcomes.

Non-contrast CT is the most commonly used imaging modality in the acute phase of the disease for reperfusion therapies such as intravenous thrombolytics and EVT, as it excludes conditions such as hemorrhage and demonstrates early ischemic findings [7,8,9]. The Alberta Stroke Program Early CT Score (ASPECTS), developed based on this imaging method, is an easy-to-apply and reliable scoring system that enables rapid assessment of early ischemic changes [10,11]. ASPECTS divides the anterior circulation territory into ten anatomical regions and allows quantitative assessment of the extent of ischemic damage [12]. Numerous studies have shown that ASPECTS < 7 is associated with poor clinical outcomes, whereas values ≥ 7 are associated with better clinical outcomes [13,14]. Therefore, ASPECTS is currently widely used, particularly for patient selection for EVT [14,15,16].

In different studies, it has been reported that infarct volume determined by magnetic resonance imaging in the acute phase shows only a moderate correlation with clinical outcome and that additional factors such as lesion localization may play a determining role in prognosis [17]. This suggests that not only the total ASPECTS but also which functional areas are represented by the anatomical localizations that constitute ASPECTS may influence clinical outcome. Although it has been proposed that there may be differences in clinical outcomes between involvement of deep and cortical regions of the brain hemispheres, the results of studies on this subject are not consistent and the available data show heterogeneity.

## 2. Materials and Methods

### 2.1. Study Design and Patient Selection

This retrospective, single-center study was conducted at Bolu Abant İzzet Baysal University Faculty of Medicine and was approved by the institutional ethics committee (approval date: 24 November 2023, decision number: 511). Due to the retrospective nature of the study, the requirement for written informed consent was waived.

Patients diagnosed with AIS between January 2020 and July 2023 who underwent non-contrast computed tomography (CT) before EVT were retrospectively screened. The study included patients who: (a) were >18 years old, (b) had ICA and/or MCA M1 segment occlusion, (c) underwent EVT, (d) had non-contrast CT images, and (e) had complete clinical and imaging data.

Patients with posterior circulation occlusion were excluded from the study. In addition, cases with insufficient imaging quality, evident chronic parenchymal lesions, or unavailable clinical follow-up data were also not included in the study. Patients with a premorbid modified Rankin Scale (mRS) score above 2 were excluded from the study.

### 2.2. Imaging Protocol

All CT examinations were performed on a 64-slice multidetector CT scanner (Revolution EVO, GE Healthcare, Milwaukee, WI, USA) using a standardized protocol: 120 kV, 320 mAs, 0.5 mm detector collimation, gantry rotation 400 ms, pitch 0.641, FOV 450–500 mm, and axial slice thickness 5 mm.

### 2.3. ASPECTS Assessment

All non-contrast brain CT images were evaluated by an experienced radiologist (with ≥5 years of experience) who was unaware of the patients’ demographic and clinical data. ASPECTS was determined based on CT imaging obtained before EVT.

ASPECTS divided the MCA territory into a total of 10 different anatomical localizations at two separate levels: the caudate nucleus, lentiform nucleus, and internal capsule, the insula, referred to as a deep cortical structure, and six cortical areas (M1–M6). One point was subtracted for the presence of early ischemic findings (hypoattenuation and/or loss of gray–white matter differentiation) in each localization. The final ASPECTS was evaluated between 0 and 10.

### 2.4. Clinical and Angiographic Data

Clinical and demographic variables, including age, sex, vascular risk factors (hypertension, diabetes mellitus, coronary artery disease, atrial fibrillation, hyperlipidemia, and smoking), and stroke side, were obtained from the hospital’s electronic patient record system. Whether the patients received intravenous thrombolytic therapy was also recorded through the same system.

Angiographic results were evaluated using the modified Thrombolysis in Cerebral Infarction (mTICI) scale, and successful reperfusion was defined as mTICI 2b–3. Clinical outcome was determined with the mRS at 3 months after stroke; an mRS ≤ 2 was accepted as a good clinical outcome and an mRS ≥ 3 as a poor clinical outcome.

### 2.5. Statistical Analysis

#### 2.5.1. Descriptive Statistics

Continuous variables were assessed for normality using the Shapiro–Wilk test. All three continuous variables (age, NIHSS, ASPECTS) were non-normally distributed (all Shapiro–Wilk *p* < 0.001) and are therefore presented as median with interquartile range (IQR: 25th–75th percentile). Between-group comparisons for continuous variables used the Mann–Whitney U test. Categorical variables are presented as frequencies and percentages, *n* (%), and compared between groups using Pearson’s chi-square test. Fisher’s exact test was used when any expected cell frequency was less than 5. Effect sizes are reported for all comparisons: rank-biserial correlation (r) for Mann–Whitney U tests and the phi coefficient (φ) for 2 × 2 chi-square tests. Effect size interpretation follows Cohen’s conventions: small (r/φ ≈ 0.10), medium (r/φ ≈ 0.30), and large (r/φ ≈ 0.50).

Binary logistic regression was used to examine the association between each ASPECTS region and poor functional outcome. In the univariable analysis, each of the 10 ASPECTS regions (M1–M6, insula, caudate, lentiform, internal capsule) was entered as a single predictor in separate models. Regions meeting a liberal significance threshold of *p* < 0.20 in univariable analysis were selected for inclusion in the multivariable model.

The multivariable logistic regression model included the selected ASPECTS regions simultaneously, adjusted for the following a priori–defined confounders: age, sex, baseline NIHSS score, occluded hemisphere (left vs. right), atrial fibrillation, diabetes mellitus, hypertension, coronary artery disease, and smoking status.

Results are presented as odds ratios (OR) for univariable models and adjusted odds ratios (aOR) for multivariable models, with 95% confidence intervals (CI). Multicollinearity was assessed using variance inflation factors (VIF), with VIF > 5 indicating problematic collinearity. Model fit was evaluated using the Hosmer-Lemeshow goodness-of-fit test (non-significant *p* indicating adequate fit), Nagelkerke R^2^ (proportion of variance explained), McFadden pseudo-R^2^, overall classification accuracy, and the area under the receiver operating characteristic curve (ROC AUC/C-statistic).

#### 2.5.2. Subgroup Analyses

Two pre-specified subgroup analyses were conducted. First, the logistic regression analysis (both univariable and multivariable) was repeated in the subgroup of patients who achieved successful recanalization, defined as modified Thrombolysis in Cerebral Infarction (mTICI) score ≥ 2b. This addresses the clinically important question of which regional infarctions predict poor outcome even when reperfusion is achieved. Second, hemisphere-stratified analyses were performed separately for right and left hemisphere occlusions. In hemisphere-stratified multivariable models, the occluded hemisphere variable was excluded from the confounder set.

#### 2.5.3. Comparative Analysis

To characterize the relationship between individual ASPECTS region involvement and total ASPECTS burden, Mann–Whitney U tests were used to compare total ASPECTS between patients with versus without infarction in each region. All statistical tests were two-tailed with a significance level of α = 0.05. Analyses were performed using Python 3 with the pandas, scipy, statsmodels, and scikit-learn libraries.

## 3. Results

A total of 283 patients were included (median age 73 years [IQR 64–80]; 57.2% female). A good functional outcome (mRS 0–2) was achieved in 147 patients (51.9%), whereas 136 (48.1%) had poor outcomes (mRS 3–6). Patients with poor outcomes were significantly older (median 74 (65–81) vs. 71 (62–79), *p* = 0.036, r = 0.14) and had higher baseline NIHSS scores (median 15 (12–19) vs. 12 (9–15), *p* < 0.001, r = 0.27). They also had lower ASPECTS (median 8 (6–9) vs. 8 (8–10), *p* < 0.001, r = 0.25). Female sex was more frequent in the poor outcome group (65.4% vs. 49.7%, *p* = 0.010, φ = 0.15). In contrast, smoking was more prevalent among patients with good outcomes (29.3% vs. 17.6%, *p* = 0.031, φ = 0.13). Successful recanalization was strongly associated with good functional outcome (94.6% vs. 79.4%, *p* < 0.001, φ = 0.22). No significant between-group differences were observed for hypertension, hyperlipidemia, atrial fibrillation, diabetes mellitus, coronary artery disease, hemisphere involved, or IV tPA administration (all *p* > 0.05) (Table 1).

The most frequently involved ASPECTS regions were the lentiform nucleus (45.9%), insula (43.8%), and caudate (32.2%). Internal capsule involvement was significantly more frequent in patients with poor outcomes compared to those with good outcomes (32.4% vs. 4.1%, *p* < 0.001, φ = 0.36). Therefore, a reliable odds ratio estimate could not be calculated for this variable. Similarly, M1 involvement was more common in the poor outcome group (26.5% vs. 10.2%, *p* < 0.001, φ = 0.20), as was insular involvement (51.5% vs. 36.7%, *p* = 0.017, φ = 0.14). No significant between-group differences were observed for M2–M6 regions, caudate, or lentiform nucleus (all *p* > 0.05) (Table 2).

In univariable analysis, M1 (OR = 3.17, 95% CI: 1.64–6.10, *p* < 0.001), insula (OR = 1.83, 95% CI: 1.14–2.94, *p* = 0.013), and internal capsule (OR = 11.24, 95% CI: 4.60–27.44, *p* < 0.001) were significantly associated with poor outcome. Six regions met the *p* < 0.20 threshold for multivariable entry. After adjustment for confounders, only M1 (aOR = 2.72, 95% CI: 1.12–6.58, *p* = 0.027) and internal capsule (aOR = 11.96, 95% CI: 4.51–31.72, *p* < 0.001) remained independently significant (Table 3).

Among the 247 patients with successful recanalization, 139 (56.3%) achieved good functional outcomes. In univariable analysis, M1 involvement (OR = 2.76, 95% CI: 1.38–5.50, *p* = 0.004), insular involvement (OR = 1.79, 95% CI: 1.07–2.99, *p* = 0.026), and internal capsule involvement (OR = 13.97, 95% CI: 5.26–37.11, *p* < 0.001) were significantly associated with poor outcome. After adjustment for age, sex, NIHSS, hemisphere, atrial fibrillation, diabetes mellitus, hypertension, coronary artery disease, and smoking, only internal capsule involvement remained independently associated with poor outcome (aOR = 18.89, 95% CI: 6.02–59.33, *p* < 0.001). Age was also independently associated with outcome (aOR = 1.03, 95% CI: 1.00–1.06, *p* = 0.041) (Table 4).

These findings suggest that internal capsule involvement is strongly associated with poor functional recovery, even in the presence of successful arterial recanalization.

In the right hemisphere subgroup (*n* = 139), M1 involvement was strongly associated with poor outcome in univariable analysis (OR = 5.30, 95% CI: 1.85–15.20, *p* = 0.002) and remained independently significant after adjustment (aOR = 7.41, 95% CI: 1.82–30.20, *p* = 0.005). Internal capsule involvement demonstrated quasi-complete separation, as nearly all affected patients experienced poor outcomes, precluding reliable estimation (Table 5B).

In the left hemisphere subgroup (*n* = 144), internal capsule involvement (OR = 6.13, 95% CI: 2.32–16.18, *p* < 0.001) and insular involvement (OR = 2.06, 95% CI: 1.06–4.01, *p* = 0.034) were significant in univariable analysis. After multivariable adjustment, only internal capsule involvement remained independently associated with poor outcome (aOR = 5.31, 95% CI: 1.80–15.68, *p* = 0.002). These findings demonstrate that the prognostic significance of specific ASPECTS regions differed between right- and left-hemisphere subgroups (Table 5C).

Total ASPECTS were significantly lower in patients with involvement of each region compared to those without involvement (all *p* < 0.001). Effect sizes were large across regions, with the highest values observed for M3 (r = 0.80), M4 (r = 0.76), and M2 (r = 0.71). M1 (r = 0.68), insula (r = 0.66), and internal capsule (r = 0.62) also demonstrated substantial associations (Table 6).

## 4. Discussion

The findings obtained in this study show that the contribution of ASPECTS regions to prognosis is not equal in patients with acute ischemic stroke due to large vessel occlusion undergoing EVT, and that some anatomical regions strongly affect clinical outcome independent of the total ASPECTS. In particular, involvement of the internal capsule and the M1 region emerged as independent determinants of poor clinical outcome in multivariable analyses. This supports that not only the extent but also the location of early ischemic changes plays a critical role in functional recovery. In this respect, our study shows that evaluating ASPECTS not only as a total score but also through its regional components may be valuable in predicting clinical outcome in patients undergoing EVT.

Involvement of the internal capsule being identified as the strongest independent predictor in this study is an anatomically and functionally expected finding. Early ischemic damage in this region, through which corticospinal tract fibers densely pass, can lead to marked motor deficits even when lesion volume is limited and directly affects the level of clinical recovery as one of the strongest determinants of motor functional outcome [18,19]. Indeed, in subgroup analyses, internal capsule involvement remained independently associated with poor clinical outcome even in patients with successful recanalization. This finding shows that achieving vessel patency does not always result in functional recovery and that preservation of strategically important anatomical regions is determinative for clinical prognosis.

Hemisphere-based analyses also revealed noteworthy results. In particular, M1 region involvement was observed as an independent predictor in occlusions with right hemisphere cortical area damage, whereas internal capsule involvement was observed as the dominant prognostic determinant in the left hemisphere. This difference may be related to interhemispheric differences in functional organization and to the hemispheric sensitivity of clinical scales (especially NIHSS). This observation may also partly reflect the known hemispheric bias of clinical scales such as the NIHSS, which may underrepresent certain right-hemisphere deficits. It is known that right hemisphere cortical damage may receive lower scores on some clinical scales despite affecting functional independence [20,21]. Therefore, localization-based evaluation may be more clinically meaningful when interpreted together with hemisphere information.

In our study, although insula involvement was found to be associated with poor outcome in univariable analyses, it lost its independence in multivariable models. In the literature, the insula region is frequently reported as a prognostically important area. However, since insula involvement often occurs as part of a more extensive MCA territory involvement, its independent effect may weaken when included in the model together with other regional variables. This situation suggests that there may be partial overlap among regional variables.

When the relationship between total ASPECTS and regional involvement was examined, it was observed that the total score was significantly lower in patients with involvement of any ASPECTS region and that effect sizes were at a high level. Nevertheless, the fact that some regions—particularly the internal capsule and M1—showed prognostic value independent of the total score suggests that localization-based weighted scoring approaches may be developed in the future.

In the literature, two different approaches stand out regarding the prognostic use of ASPECTS assessment. Some studies report that the cumulative ASPECTS remains a stable and reliable marker for predicting clinical outcome and that localization-weighted scoring approaches do not provide a significant advantage over the total score [22]. In contrast, many studies have shown that the prognostic contribution of ASPECTS regions is not homogeneous; however, they emphasize that the results are heterogeneous and vary across studies regarding which anatomical regions more strongly affect clinical outcome [17,23,24]. The findings of our study support this second approach and show that involvement of the internal capsule and the M1 region is strongly associated with clinical outcome, independent of the total ASPECTS.

In the study published in 2015 by Beare et al., the M2 region in particular emerged as the area showing the strongest association with functional outcome, followed by the M5 and insula regions [25]. In a non-contrast CT–based study conducted at two centers, including patients who underwent endovascular thrombectomy due to anterior circulation acute ischemic stroke, it was shown that the contribution of ASPECTS regions to clinical outcome was not equal. In that study, involvement of the caudate nucleus, M4, and insula was significantly associated with poor clinical outcome at three months (mRS 3–6), whereas involvement of the M1 region was reported to reduce the likelihood of poor clinical outcome [23]. In another non-contrast CT–based study published in 2016, in which data from a total of 1115 patients were analyzed, involvement of the caudate nucleus, lentiform nucleus, insula, and M5 regions was identified as an independent predictor of poor clinical outcome. Based on these findings, a score called “simplified ASPECTS (sASPECTS),” including only these four regions, was developed, and this score was shown to predict clinical outcome with accuracy similar to the classical 10-point ASPECTS; thus, it demonstrated that infarct localization is determinative for prognosis independent of total lesion burden [26].

In one of the recent studies supporting the prognostic value of regional ASPECTS contribution, Zou et al. examined regional infarct volume ratios based on artificial intelligence–assisted perfusion analysis in patients undergoing EVT and showed that involvement of the internal capsule and the M6 region were independent predictors of poor clinical outcome. In addition, it was reported that as the within-region infarct volume ratio increased, the likelihood of poor prognosis increased markedly [27,28].

Our study, unlike previous studies, evaluated not only the regional ASPECTS contribution but also separately assessed the prognostic effect of regional involvement in the successful recanalization subgroup and in hemisphere-based analyses. This multilayered analysis approach is important in demonstrating that the effect of some ASPECTS regions on prognosis remains consistently sustained across different clinical scenarios.

This study has several limitations. First, since the study has a retrospective and single-center design, the possibility of selection bias cannot be completely excluded and the generalizability of the findings may be limited. ASPECTS assessment was performed by a single observer; although the evaluator was experienced, the lack of interobserver agreement analysis may be considered a limitation. The evaluation was based only on non-contrast CT imaging; the lack of combined analysis of lesion volume and penumbra area with advanced imaging methods (perfusion CT or diffusion MRI) may have led to additional prognostic information being overlooked. In addition, the relatively limited sample size in some subgroup analyses led to wide confidence intervals, especially in hemisphere-stratified models. Because the analyses were hypothesis-driven, correction for multiple comparisons was not applied. The absence of correction for multiple comparisons also requires cautious interpretation of borderline significant results. Future studies incorporating calibration plots may provide additional insight into model calibration and predictive performance.

Advanced MRI-based techniques may provide more comprehensive and complementary information about early tissue injury in the acute phase of ischemic stroke. In particular, quantitative susceptibility mapping (QSM) has been shown to detect iron deposition, microvascular injury, and the risk of hemorrhagic transformation, which may further refine imaging-based prognostic models in future studies [29,30].

When these findings are evaluated in terms of clinical practice, they suggest that in patients who are candidates for EVT, not only the total ASPECTS but also the regional distribution of early ischemic changes may be useful to report systematically. In particular, early recognition of internal capsule and M1 region involvement may provide additional value in predicting clinical outcome and in informing patients’ relatives. The development of regionally weighted ASPECTS approaches through future multicenter and prospective studies may increase the accuracy of imaging-based prognostic models. Potential collinearity between regional ASPECTS variables and the total ASPECTS was considered during the analysis; therefore, the total ASPECTS was not included in the multivariable models to avoid structural dependence. Including total ASPECTS together with regional variables would introduce structural overlap, as the regional components directly contribute to the cumulative score.

## 5. Conclusions

In patients with acute ischemic stroke due to large vessel occlusion undergoing endovascular thrombectomy, the contribution of ASPECTS regions to clinical outcome is not homogeneous. In particular, involvement of the internal capsule and the M1 region was identified as a strong determinant of poor clinical outcome independent of the total ASPECTS. It is noteworthy that internal capsule involvement preserves its prognostic value even in patients with successful recanalization. However, patient selection for EVT based solely on lesion localization does not appear to be sufficient; nevertheless, this finding may be particularly recommended to be considered in prognostic evaluation in patients with internal capsule involvement. Further multicenter studies are needed to clarify the role of regional ASPECTS assessment in clinical decision processes.

## Figures and Tables

**Table 1:** Demographic and Clinical Characteristics by Functional Outcome.

Variable	Total (*n* = 283)	Good Outcome (*n* = 147)	Poor Outcome (*n* = 136)	*p*	Effect Size
Age, median (IQR)	73 (64–80)	71 (62–79)	74 (65–81)	**0.036**	r = 0.14
NIHSS, median (IQR)	14 (10–17)	12 (9–15)	15 (12–19)	**<0.001**	r = 0.27
ASPECTS, median (IQR)	8 (7–9)	8 (8–10)	8 (6–9)	**<0.001**	r = 0.25
Female sex, *n* (%)	162 (57.2)	73 (49.7)	89 (65.4)	**0.010**	φ = 0.15
Left hemisphere, *n* (%)	144 (50.9)	75 (51.0)	69 (50.7)	1.000	φ = 0.00
Hypertension, *n* (%)	185 (65.4)	90 (61.2)	95 (69.9)	0.162	φ = 0.08
Hyperlipidemia, *n* (%)	36 (12.7)	21 (14.3)	15 (11.0)	0.520	φ = 0.04
Atrial fibrillation, *n* (%)	117 (41.3)	59 (40.1)	58 (42.6)	0.758	φ = 0.02
Diabetes mellitus, *n* (%)	88 (31.1)	45 (30.6)	43 (31.6)	0.957	φ = 0.00
Coronary artery disease, *n* (%)	79 (28.0)	44 (30.1)	35 (25.7)	0.490	φ = 0.04
Smoking, *n* (%)	67 (23.7)	43 (29.3)	24 (17.6)	**0.031**	φ = 0.13
IV tPA, *n* (%)	63 (22.3)	31 (21.1)	32 (23.5)	0.726	φ = 0.02
Successful recanalization, *n* (%)	247 (87.3)	139 (94.6)	108 (79.4)	**<0.001**	φ = 0.22

Note. Continuous variables were non-normally distributed (Shapiro–Wilk *p* < 0.001 for all) and are presented as median (IQR); compared using Mann–Whitney U test. Categorical variables: *n* (%); compared using Pearson’s chi-square test. Fisher’s exact test was not required (all expected cell frequencies ≥ 5). One patient had missing CAD data (treated as missing; N = 282 for CAD). Effect sizes: rank-biserial r for continuous variables, phi coefficient (φ) for categorical variables. Interpretation: r/φ ≈ 0.10 (small), ≈0.30 (medium), ≈0.50 (large) per Cohen (1988). Bold *p* values: *p* < 0.05 (two-tailed). Good outcome = mRS 0–2; Poor outcome = mRS 3–6.

**Table 2:** ASPECTS Region Involvement Frequency and Association with Outcome.

ASPECTS Region	Total *n* (%)	Good Outcome *n* (%)	Poor Outcome *n* (%)	*p*	φ
**M1**	51 (18.0)	15 (10.2)	36 (26.5)	**<0.001**	0.20
**M2**	40 (14.1)	15 (10.2)	25 (18.4)	0.071	0.11
**M3**	19 (6.7)	6 (4.1)	13 (9.6)	0.109	0.10
**M4**	10 (3.5)	5 (3.4)	5 (3.7)	1.000	0.00
**M5**	17 (6.0)	9 (6.1)	8 (5.9)	1.000	0.00
**M6**	21 (7.4)	12 (8.2)	9 (6.6)	0.788	0.02
**Insula**	124 (43.8)	54 (36.7)	70 (51.5)	**0.017**	0.14
**Caudate**	91 (32.2)	41 (27.9)	50 (36.8)	0.142	0.09
**Lentiform**	130 (45.9)	64 (43.5)	66 (48.5)	0.470	0.04
**Internal Capsule**	50 (17.7)	6 (4.1)	44 (32.4)	**<0.001**	0.36

Note: Bold values indicate statistical significance (*p* < 0.05). *n* (%) = patients with early ischemic changes. Chi-square test or Fisher’s exact test (M4: expected frequency < 5). φ = phi coefficient. Interpretation: φ ≈ 0.10 (small), ≈0.30 (medium), ≈0.50 (large). Multiple testing correction not applied (hypothesis-driven); interpret borderline values cautiously, given 10 comparisons. Bold: *p* < 0.05.

**Table 3:** Logistic Regression: ASPECTS Regions Predicting Poor Outcome (mRS 3–6).

Variable	Univariable OR (95% CI)	*p*	Multivariable aOR (95% CI)	*p*	VIF
**ASPECTS Regions**					
M1	3.17 (1.64–6.10)	**<0.001**	2.77 (1.14–6.74)	**0.025**	1.51
M2	1.98 (1.00–3.94)	0.051	1.02 (0.35–3.00)	0.968	1.98
M3	2.48 (0.92–6.73)	0.074	1.93 (0.52–7.16)	0.328	1.53
M4	1.08 (0.31–3.83)	0.900	—	—	—
M5	0.96 (0.36–2.56)	0.932	—	—	—
M6	0.80 (0.32–1.96)	0.621	—	—	—
Insula	1.83 (1.14–2.94)	**0.013**	1.22 (0.68–2.17)	0.502	1.22
Caudate	1.50 (0.91–2.48)	0.111	1.00 (0.53–1.87)	0.998	1.21
Lentiform	1.22 (0.77–1.95)	0.400	—	—	—
Internal Capsule	11.24 (4.60–27.44)	**<0.001**	11.72 (4.41–31.18)	**<0.001**	1.22
**Confounders**					
Age	—	—	1.02 (1.00–1.05)	0.090	1.35
Female sex	—	—	1.40 (0.76–2.59)	0.284	1.32
NIHSS	—	—	1.04 (0.99–1.09)	0.132	1.13
Left hemisphere	—	—	0.82 (0.46–1.47)	0.510	1.19
AF	—	—	0.92 (0.51–1.67)	0.794	1.22
DM	—	—	0.89 (0.49–1.61)	0.692	1.07
Hypertension	—	—	1.37 (0.71–2.66)	0.351	1.35
CAD	—	—	0.78 (0.42–1.45)	0.437	1.09
Smoking	—	—	0.80 (0.40–1.62)	0.542	1.27

Note. OR = odds ratio; aOR = adjusted odds ratio; CI = confidence interval; VIF = variance inflation factor; Dependent variable: poor outcome (mRS 3–6). Univariable: each region tested independently. Multivariable (N = 282): regions with univariable *p* < 0.20 adjusted for age, sex, NIHSS, hemisphere, AF, DM, hypertension, CAD, and smoking. All VIF < 2.0. Model fit: Nagelkerke R^2^ = 0.296; accuracy = 71.3%; ROC AUC = 0.768. The Hosmer-Lemeshow test was significant (χ^2^(8) = 25.03, *p* = 0.002), suggesting suboptimal calibration. However, this test is known to be sensitive to the number of grouping deciles; when computed with g = 12, the result was non-significant (χ^2^(10) = 16.61, *p* = 0.084). Given the adequate discrimination and explained variance, the model was considered acceptable for identifying independent predictors. Regions with univariable *p* ≥ 0.20 not entered into multivariable model (—). Bold: *p* < 0.05.

**Table 4:** Logistic Regression in Successful Recanalization Subgroup (mTICI ≥ 2b; N = 247).

Variable	Univariable OR (95% CI)	*p*	Multivariable aOR (95% CI)	*p*
**ASPECTS Regions**				
M1	2.76 (1.38–5.50)	**0.004**	2.36 (0.90–6.17)	0.080
M2	1.88 (0.91–3.87)	0.087	1.20 (0.39–3.76)	0.748
M3	2.26 (0.80–6.43)	0.126	2.08 (0.52–8.25)	0.298
M4	1.03 (0.27–3.93)	0.965	—	—
M5	1.00 (0.36–2.78)	0.998	—	—
M6	0.85 (0.33–2.15)	0.726	—	—
Insula	1.79 (1.07–2.99)	**0.026**	1.12 (0.59–2.13)	0.732
Caudate	1.50 (0.87–2.58)	0.141	0.75 (0.37–1.55)	0.441
Lentiform	1.32 (0.79–2.18)	0.286	—	—
Internal Capsule	13.97 (5.26–37.11)	**<0.001**	18.89 (6.02–59.33)	**<0.001**
**Confounders**				
Age	—	—	1.03 (1.00–1.06)	**0.041**
Female sex	—	—	1.46 (0.73–2.92)	0.280
NIHSS	—	—	1.04 (0.99–1.09)	0.172
Left hemisphere	—	—	0.81 (0.43–1.54)	0.526
AF	—	—	0.99 (0.51–1.91)	0.973
DM	—	—	0.90 (0.47–1.73)	0.756
Hypertension	—	—	1.75 (0.83–3.67)	0.141
CAD	—	—	0.71 (0.36–1.42)	0.336
Smoking	—	—	1.00 (0.46–2.16)	0.997

Note. Good outcome *n* = 139, poor outcome *n* = 108. Multivariable model: regions with univariable *p* < 0.20 + confounders (age, sex, NIHSS, hemisphere, AF, DM, hypertension, CAD, smoking). Model fit: Nagelkerke R^2^ = 0.336; Hosmer-Lemeshow *p* = 0.118; ROC AUC = 0.791; accuracy = 73.2%. Bold: *p* < 0.05.

**Table 5:** Logistic Regression Stratified by Occluded Hemisphere.

A. Univariable Analysis by Hemisphere				
**ASPECTS Region**	**Right (*n* = 139) OR (95% CI)**	***p***	**Left (*n* = 144) OR (95% CI)**	***p***
M1	5.30 (1.85–15.20)	**0.002**	2.13 (0.90–5.03)	0.087
M2	2.35 (0.76–7.28)	0.138	1.81 (0.75–4.34)	0.187
M3	4.08 (0.82–20.40)	0.087	1.69 (0.46–6.26)	0.432
M4	1.46 (0.31–6.78)	0.629	0.54 (0.05–6.05)	0.615
M5	1.08 (0.30–3.91)	0.906	0.81 (0.17–3.74)	0.784
M6	0.75 (0.23–2.48)	0.636	0.86 (0.22–3.35)	0.830
Insula	1.62 (0.82–3.19)	0.165	2.06 (1.06–4.01)	**0.034**
Caudate	1.58 (0.75–3.33)	0.228	1.45 (0.73–2.87)	0.285
Lentiform	1.20 (0.61–2.36)	0.592	1.25 (0.65–2.40)	0.509
Internal Capsule	N/E †	N/E	6.13 (2.32–16.18)	**<0.001**
B. Multivariable Model—Right Hemisphere (*n* = 139)				
**Variable**	**aOR (95% CI)**	***p***	**VIF**	
M1	7.41 (1.82–30.20)	**0.005**	1.52	
M2	0.47 (0.06–3.98)	0.490	3.03	
M3	4.87 (0.38–62.53)	0.224	2.52	
Insula	1.07 (0.48–2.40)	0.866	1.24	
Age	1.02 (0.99–1.05)	0.241	1.21	
Female sex	1.37 (0.58–3.20)	0.473	1.37	
NIHSS	1.04 (0.98–1.11)	0.148	1.05	
AF	0.64 (0.28–1.44)	0.277	1.24	
DM	0.91 (0.41–2.01)	0.809	1.11	
Hypertension	1.80 (0.69–4.68)	0.228	1.34	
CAD	0.52 (0.22–1.21)	0.128	1.11	
Smoking	1.25 (0.47–3.32)	0.650	1.18	
C. Multivariable Model—Left Hemisphere (*n* = 144)				
**Variable**	**aOR (95% CI)**	***p***	**VIF**	
M1	1.38 (0.41–4.62)	0.604	1.58	
M2	1.34 (0.39–4.53)	0.641	1.60	
Insula	1.65 (0.73–3.74)	0.232	1.25	
Internal Capsule	5.31 (1.80–15.68)	**0.002**	1.20	
Age	1.01 (0.97–1.04)	0.647	1.49	
Female sex	1.70 (0.71–4.07)	0.235	1.41	
NIHSS	1.06 (0.98–1.15)	0.167	1.14	
AF	1.20 (0.51–2.82)	0.682	1.26	
DM	0.96 (0.40–2.31)	0.929	1.09	
Hypertension	1.05 (0.43–2.58)	0.914	1.41	
CAD	0.90 (0.36–2.23)	0.820	1.12	
Smoking	0.54 (0.19–1.50)	0.239	1.49	

Note. Bold values indicate statistical significance (*p* < 0.05). OR = odds ratio; CI = confidence interval; N/E = not estimable. † Quasi-complete separation: nearly all affected patients had poor outcomes, preventing reliable estimation. The internal capsule in the right hemisphere subgroup showed quasi-complete separation (nearly all affected patients had poor outcomes), preventing reliable OR estimation. Right hemisphere: good outcome *n* = 72, poor outcome *n* = 67. Left hemisphere: good outcome *n* = 75, poor outcome *n* = 69. Bold *p* values indicate statistical significance (*p* < 0.05. Bold: *p* < 0.05. Adjusted for age, sex, NIHSS, AF, DM, hypertension, CAD, and smoking (hemisphere excluded). Model fit: Nagelkerke R^2^ = 0.205; ROC AUC = 0.712; accuracy = 60.4%. Adjusted for age, sex, NIHSS, AF, DM, hypertension, CAD, and smoking. Model fit: Nagelkerke R^2^ = 0.279; ROC AUC = 0.775; accuracy = 72.7%.

**Table 6:** Total ASPECTS Comparison by Regional Infarction.

Region	*n*	Affected ASPECTS	*n*	Preserved ASPECTS	Test	*p*	r
M1	51	7 (6–7)	232	8 (8–10)	U = 1884	**<0.001**	0.68
M2	40	6 (6–7)	243	8 (8–10)	U = 1430	**<0.001**	0.71
M3	19	6 (6–6)	264	8 (7–9)	U = 509	**<0.001**	0.80
M4	10	6 (4–7)	273	8 (7–9)	U = 330	**<0.001**	0.76
M5	17	6 (4–7)	266	8 (7–9)	U = 716	**<0.001**	0.68
M6	21	6 (4–7)	262	8 (7–9)	U = 930	**<0.001**	0.66
Insula	124	7 (6–8)	159	9 (8–10)	U = 3331	**<0.001**	0.66
Caudate	91	7 (6–8)	192	9 (8–10)	U = 3645	**<0.001**	0.58
Lentiform	130	8 (7–8)	153	9 (8–10)	U = 5092	**<0.001**	0.49
Internal Capsule	50	6 (6–8)	233	8 (8–10)	U = 2206	**<0.001**	0.62

Note: Explanation for bold values has been added. Median (IQR). All non-normally distributed (Shapiro–Wilk *p* < 0.001). Mann–Whitney U test. r = rank-biserial correlation. Interpretation: r ≈ 0.10 (small), ≈0.30 (medium), ≈0.50 (large). Bold: *p* < 0.001.

## Data Availability

The data that support the findings of this study are available from the corresponding author upon reasonable request.
